# Improving GHB withdrawal with baclofen: study protocol for a feasibility study for a randomised controlled trial

**DOI:** 10.1186/s13063-016-1593-9

**Published:** 2016-09-27

**Authors:** Anne Lingford-Hughes, Yash Patel, Owen Bowden-Jones, Mike J. Crawford, Paul I. Dargan, Fabiana Gordon, Steve Parrott, Tim Weaver, David M. Wood

**Affiliations:** 1 Central North West London NHS Foundation Trust’s Club Drug Clinic, 69 Warwick Rd, London, SW5 9HB UK; 2 Centre for Psychiatry, Division of Brain Sciences, Imperial College London, Burlington Danes Building, Hammersmith Hospital site, Du Cane Rd, London, W12 0NN UK; 3 Guy’s and St. Thomas NHS Foundation Trust, London, UK; 4 Faculty of Life Sciences and Medicine, King’s College, London, UK; 5 Statistical Advisory Service, School Of Public Health, Imperial College London, London, UK; 6 Department of Health Sciences, The University of York, York, UK; 7 Department of Mental Health, Social Work and Integrative Medicine, Middlesex University, London, UK

**Keywords:** GHB, Gamma-hydroxybutyrate, GBL, Gamma-butyrolactone, GHB/GBL withdrawal, Baclofen, Benzodiazepine, GABA_B_, GHB/GBL dependence

## Abstract

**Background:**

GHB (gamma-hydroxybutyrate) and its pro-drugs GBL (gamma-butyrolactone) and 1,4-butanediol (1,4-BD) are central nervous system depressants whose street names include ‘G’ and ‘liquid ecstasy’. They are used recreationally predominately for their stimulant and pro-sexual effects or for sedation to help with sleep and/or to ‘come down’ after using stimulant recreational drugs. Although overall population prevalence is low (0.1 %), in some groups such as men who have sex with men, GHB/GBL use may reach 20 %. GHB/GBL dependence may be associated with severe withdrawal with individuals presenting either acutely to emergency departments or to addiction services for support. Benzodiazepines are currently prescribed for GHB/GBL detoxification but do not prevent all complications, such as behavioural disinhibition, that may require hospitalisation or admission to a high dependency/intensive care unit. The GABA_B_ receptor mediates most effects of GHB/GBL and the GABA_B_ agonist, baclofen, has shown promise as an adjunct to benzodiazepines in reducing withdrawal severity when prescribed both during withdrawal and as a 2-day ‘preload’ prior to detoxification. The key aim of this feasibility study is provide information about recruitment and characteristics of the proposed outcome measure (symptom severity, complications including delirium and treatment escalation) to inform an application for a definitive randomised placebo controlled trial to determine the role of baclofen in the management of GHB/GBL withdrawal and whether starting baclofen 2 days earlier improves outcomes further.

**Methods/design:**

This is a prospective, randomised, double-blind, placebo-controlled feasibility study that will recruit participants (aged over 18 years) who are GHB/GBL-dependent and wish to undergo planned GHB/GBL detoxification or are at risk of acute withdrawal and are inpatients requiring unplanned withdrawal. We aim to recruit 88 participants: 28 unplanned inpatients and 60 planned outpatients.

During detoxification we will compare baclofen 10 mg three times a day with placebo as an adjunct to the usual benzodiazepine regimen. In the planned outpatient arm, we will also compare a 2-day preload of baclofen 10 mg three times a day with placebo. Ratings of GHB/GBL withdrawal, sleep, depression, anxiety as well as GHB/GBL use will be collected. The main data analyses will be descriptive about recruitment and characterising the impact of adding baclofen to the usual benzodiazepine regimen on measures and outcomes of GHB/GBL withdrawal to provide estimates of variability and effect size. A qualitative approach will evaluate research participant and clinician acceptability and data collected to inform cost-effectiveness.

**Discussion:**

This feasibility study will inform a randomised controlled trial to establish whether adding baclofen to a benzodiazepine regimen reduces the severity and complications of GHB/GBL withdrawal.

**Trial registration:**

ISRCTN59911189. Registered 14 October 2015. Protocol: v3.1, 1 February 2016

## Background

### GHB/GBL use and dependence

GHB (gamma-hydroxybutyrate) and its related analogues GBL (gamma-butyrolactone) and 1,4-butanediol (1,4-BD) are central nervous system depressants and their street names include ‘G’ and ‘liquid ecstasy’ [1, 2]. GBL and 1,4-BD are converted to GHB after ingestion and, therefore, all three drugs have similar pharmacological actions and profiles [1, 2]. There is limited use of 1,4-BD in the UK and, therefore, hereafter we will use the term GHB/GBL to refer to these compounds. They are used recreationally predominately for their stimulant and pro-sexual effects, although some individuals use them for their sedative effects and/or to help ‘come down’ after using stimulant recreational drugs [3–5]. Although overall population prevalence of use is low, the Crime Survey for England and Wales reported that use significantly rose from 0.0 % in 2010/11 to 0.1 % in 2011/12; subsequent surveys did not collect GHB/GBL data [6]. However, the use of these drugs is more common in a number of subpopulation groups. In particular, in populations such as clubbers and men who have sex with men (MSM), lifetime prevalence of GHB/GBL use ranges from 3.9 to 14.3 %, with last-month prevalence of use of up to 4.6 % [7]. Almost a quarter of those surveyed in ‘gay-friendly’ South London dance clubs in July 2011 reported GHB/GBL use that night, second to mephedrone at 41 % [8]. Similarly, attendees at two London sexual health clinics (December 2013 to March 2104) reported lifetime prevalence of use of GHB at 19 % and GBL at 13 % [9].

Reports from the UK’s Advisory Council on Misuse of Drugs [10] and European Monitoring Centre for Drugs and Drug Addiction (EMCDDA) [7] have highlighted the potential for both significant acute toxicity and also dependence associated with GHB/GBL. The latest data from Public Health England reports that the numbers of people presenting to treatment services with problems with GHB/GBL continue to rise from 18 (2 % of new presentations involving ‘club drugs’ (methamphetamine, mephedrone, ketamine, ecstasy, and GHB/GBL)) of those seeking treatment in 2005–2006 to 249 (5 %) in 2013–2014 [11] and the number of deaths implicating GHB are also rising from none in 1993 to 12–20/year in 2008–2012 [12]. Our clinical experience is consistent with this. Presentations with acute GHB/GBL toxicity to the emergency department (ED) and Clinical Toxicology Service (one of the sites in this study) at Guy’s and St. Thomas’s Hospital Foundation Trust (GSTT) increased from 158 in 2006 to 270 in 2010 [13]; and there has been an increase in the numbers of individuals seeking help from services such as Antidote (supporting the lesbian, gay, bisexual and transgender (LGBT) community) and the Club Drug Clinic, Central North West London NHS Foundation Trust (CNWL) for GHB-related problems. Antidote has seen an increase in GHB-related problems from 1.7 % (*n* = 3) of total referrals in 2005 to 57 % (*n* = 317) in 2010 and 44 % (*n* = 334) in 2013–2014.

GHB/GBL use is associated with a risk of physical dependence and a potentially life-threatening withdrawal syndrome, unlike other club drugs such as stimulants which do not have a pronounced withdrawal syndrome [14]. Therefore, a disproportionate number of GHB/GBL-dependent users compared with other club drug users require help for a potentially life-threatening withdrawal syndrome. Management of GHB/GBL withdrawal or detoxification can present challenges to clinicians and dependent users due to the rapidity of onset of the withdrawal and the severe clinical features that can occur. Regular use of GHB/GBL (typically multiple times per day every day over a period of at least a few months) can lead to dependence with dependent users typically using the drug every 1–4 h to prevent the onset of withdrawal [15–18]. Withdrawal symptoms occur typically within hours following last use due to GHB/GBL’s rapid elimination (T_1/2_ is approximately 27 min) and may also occur during recovery from acute intoxication (overdose) [2, 11, 18, 19]. A proportion of the patients with GHB dependence may, therefore, use alcohol or other drugs such as benzodiazepines, ‘Z drugs’ (e.g. zopiclone) and/or baclofen to self-manage withdrawal symptoms, insomnia and cravings. Typically these are used to help increase the time between dosing overnight to enable them to sleep. The clinical features of GHB withdrawal are similar to alcohol withdrawal but often with more rapid onset. GHB withdrawal can also resemble acute stimulant toxicity and coingestion of other drugs. Identification of GHB dependence and withdrawal can be complicated by a lack of awareness by nonspecialist centres, particularly in emergency cases [20].

### GHB/GBL withdrawal and its treatment

GHB/GBL withdrawal has many similar features to alcohol withdrawal, including tremor, sweating, anxiety, agitation and confusion; however, it is generally more severe, has a more rapid onset and more prominent neuropsychiatric features such as delirium and psychosis [2, 15, 18]. In common with alcohol withdrawal, benzodiazepines have been the pharmacotherapeutic mainstay for GHB/GBL withdrawal [18]. However, our clinical experience suggests that using benzodiazepines alone may be insufficient, with up to 50 % of individuals presenting to our ED and initially 4 % of those in our outpatient clinic requiring acute medical care [21, 22]. This latter group had either developed significant delirium and/or worsening of their initial acute delirium on presentation that required escalation of treatment, including admission to intensive care and use of additional sedatives (e.g. barbiturates or propofol) with the associated intubation and mechanical ventilation for airway support as described [15]. Acute delirium, agitation and/or psychosis results in further risks to the individual, as well as to the staff treating them due to the severity of their agitation and violent behaviour. There has been one death reported as a ‘complication of GHB/GBL withdrawal’. The individual in this case was treated with benzodiazepines for 12 days, developed pneumonia and suffered a cardiac arrest and we feel that the significance of the GHB dependency/withdrawal in the death is unclear and it is likely that the death is not directly related to dependence/withdrawal [23]. Despite the severity of GHB/GBL withdrawal and concerns about how to best manage unplanned and planned detoxification, at this time there is no systematic evidence base on which to base a nationally or internationally agreed treatment protocol. One review produced an algorithm based on the amount of use and the presence of delirium [24]. For those with severe dependence (more than three doses of GHB/GBL per 24 h or over 30 g GHB or/over 15 g GBL) and ‘medical complications’, admission for detoxification and supportive medical care with high-dose diazepam (150–200 mg/24 h) was suggested. Pentobarbital (barbiturate) in the intensive care unit (ICU) was suggested for treating those presenting with delirium. However, it was not clear how long to use pentobarbital for or what to do in those who do not respond to this treatment escalation. For those without delirium, diazepam reducing from 80 to 150 mg per day over 7 days whilst an inpatient was proposed. For those using less GHB/GBL, outpatient management with diazepam reducing from 20 to 40 mg per day over 7 days was suggested. Given the complexity, rapidity of onset of symptoms and complications of withdrawal, such as acute severe delirium, it is important that daily outpatient supervision of GHB/GBL withdrawal occurs in a setting in which admission to an acute hospital is possible should complications arise [25].

Various other pharmacological approaches have been investigated. One trial compared lorazepam and pentobarbital in inpatients for GHB/GBL withdrawal; however, this was not completed due to the inability to recruit sufficient GHB-dependent individuals [26]. Whilst pentobarbital is safe to use in hospital, due to the potential risk of coma and lethal toxicity it is not appropriate for use in community/outpatient detoxification. A Dutch pilot uncontrolled study reported that a reducing regimen of GHB/GBL successfully treated withdrawal and prevented complications, such as delirium, in 23 GHB/GBL-dependent inpatients [27]. GHB in the pharmaceutical preparation sodium oxybate is licensed in the UK for the treatment of narcolepsy associated with cataplexy in specialist sleep services. Current UK prescribing, storage and administration controls (due to the legal status of GHB under the UK Misuse of Drugs Act, 1971) are likely to be practical considerations that limit its usefulness in managing GHB/GBL withdrawal (due to delays in treatment administration). Antipsychotic agents may lower the seizure threshold increasing the risk of GHB withdrawal-related seizures. However, they may also interact with GHB’s effects on the dopaminergic system increasing the risk of neuroleptic malignant syndrome [17, 28].

In summary, a reducing regimen of benzodiazepines alone for GHB/GBL withdrawal is currently considered ‘standard’ treatment and as such is the current ‘best practice’; although in a significant proportion of individuals benzodiazepines alone may be insufficient. The limited research almost always suggests benzodiazepines as the core treatment, with a range of other options having been suggested as helpful of which baclofen has strong pharmacological validity (see next section).

### GHB/GBL withdrawal and the GABA_B_ system

In addition to its activity at endogenous GHB receptors GHB acts at GABA_B_ receptors, which play a key role in withdrawal since GABA_B_ antagonists precipitate withdrawal in GHB-dependent nonhuman primates [1, 29] and can block GHB-induced respiratory depression [30]. Benzodiazepines work through GABA_A_ receptors; however, GHB has limited activity at these receptors, likely explaining their apparently incomplete clinical effectiveness in acute GHB withdrawal. The GABA_B_ agonist baclofen has, therefore, been used on an unlicensed named-patient basis as an adjunct to benzodiazepines to manage GHB/GBL withdrawal by the authors and others [18, 31]. Baclofen is currently only licensed in the UK for the management of muscle spasticity in multiple sclerosis and other conditions.

An uncontrolled case series in 19 GHB/GBL-dependent patients, all of whom except for 2 underwent outpatient treatment, reported that baclofen (10 mg three times a day) in addition to high-dose diazepam during the initial 4–5 days of GHB/GBL detoxification, resulted in no transfers to ICU and several patients commented that baclofen was helpful [18]. Furthermore, the experience of clinical toxicologists and addiction specialists in the UK, including those involved in this study, has been that baclofen is helpful in reducing the complications from GHB/GBL withdrawal. Consequently, the recent update of the British Association for Psychopharmacology’s addiction guidelines [25] suggested using baclofen (10 mg three times a day) as an adjunct to benzodiazepines for GHB/GBL withdrawal.

Furthermore, our clinical experience of GHB/GBL withdrawal in outpatient settings has been that patients who slowly reduce their GHB/GBL use describe significant anxiety and cravings during the few days before commencing medically assisted detoxification. For some, this acted as a deterrent to attending for treatment and a trigger for using higher doses of GHB/GBL. Consequently, we have initially used baclofen 2 days prior to stopping GHB/GBL in a small number of patients. There was very positive feedback from patients regarding the benefit, particularly in terms of reduced cravings and helping to stabilise pre-detoxification GHB use. The use of baclofen in our outpatient clinical service prior to initiating medically assisted detoxification has now become more widespread. The 2 days of pre-detoxification ‘preloading’ of baclofen at a standard dose of 10 mg three times a day has not resulted in any clinical incidents in relation to baclofen. This preloading with baclofen has previously not been formally studied to determine the usefulness of baclofen itself, neither has whether some of the reported benefits relate more to engagement with drug treatment services pre-detoxification than to baclofen itself.

Baclofen has been licensed in the UK for many years for the treatment of spasticity (maximum, 100 mg per day) and can be safely prescribed to a wide range of patients (see Summary of Product Characteristics (SPC), https://www.medicines.org.uk/emc/medicine/23850). Our clinical experience is that it is associated with few side effects. Whilst a withdrawal state is recognised for baclofen, this is unlikely to occur with its use in acute GHB withdrawal due to the short duration (7 to 10 days) of baclofen use in this indication. We suggest that the risk of complications from baclofen withdrawal is considerably less than that of inadequately managed GHB/GBL withdrawal [15, 18]. Nevertheless, whilst the use of baclofen holds promise, there are potential adverse effects on cardiovascular, neurological and respiratory systems so controlled data is urgently required to determine its efficacy and safety in GHB/GBL withdrawal. Optimising outpatient treatment to reduce the risk of complications and hospital admission is important since many individuals decline admission [17, 25]. Despite this complexity and its impact on, and cost to, the individual and the NHS, there is limited knowledge about how to best treat people in planned or unplanned GHB/GBL withdrawal.

### Primary and secondary objectives

The primary objective of this study is to investigate the feasibility of recruiting GHB/GBL-dependent patients and to characterise the impact of adding baclofen to a standard benzodiazepine regimen for the management of GHB/GBL withdrawal in both outpatient (community) and inpatient general hospital settings.

There are several other secondary objectives. These are to examine:

- Withdrawal symptoms and complications, such as delirium and the requirement for treatment escalation, during detoxification in two populations: those presenting to an ED requiring immediate acute withdrawal management (unplanned) and those presenting to a specialist outpatient Club Drug Clinic requiring GHB/GBL detoxification (planned)
- Whether, as part of a planned detoxification, starting baclofen 2 days prior to stopping GHB/GBL confers additional benefits in our proposed primary outcome measures (symptom severity, complications such as delirium and requirement for treatment escalation)
- Recruitment rate monitoring and manage any difficulties
- The impact of GHB/GBL withdrawal on secondary outcome measures (anxiety, depression, sleep, quality of life)
- The impact of study participation on GHB/GBL use up to 1 month post randomisation and other alcohol/drug use
- The views of the research participants and staff about the acceptability of the study design
- Preliminary information gathered regarding costs of GHB/GBL withdrawal and its management to develop a full economic analysis in a definitive trial

## Methods/design

This is a prospective, randomised, double-blind, placebo-controlled trial to assess the feasibility of undertaking a definitive trial investigating the efficacy of baclofen in treating GHB/GBL withdrawal, both in planned and unplanned withdrawal using both quantitative and qualitative approaches. The optimal recruitment rate and strategies and characteristics of the proposed primary outcome measures (symptom severity, complications, and requirement for treatment escalation) will be assessed. Ethical approval has been obtained from National Research Ethics Service Committee London – Dulwich and the EUDRACT number is 2013-005319-28.

### Research settings

The study will recruit from two services where individuals present for treatment of GHB/GBL withdrawal. The CNWL Club Drug Clinic, based in Central London, UK is an outpatient clinic where individuals are referred or self-present for treatment for GHB/GBL dependence and may undergo planned detoxification as part of their treatment package, and secondly, to a specialist Clinical Toxicology Service at Guy’s and St. Thomas’ NHS Foundation Trust, London, UK for those who present with unplanned withdrawal requiring immediate management. For planned detoxification group recruitment, we have produced a trial poster and short information sheet for use at our linked clinical services to ensure an adequate appropriate recruitment rate. We will display this poster at such clinical services and staff will be encouraged to provide a copy of the short information sheet for planned withdrawal.

### Participants

#### Inclusion criteria

Any individual who is over 18 years old, is either in active GHB/GBL withdrawal or has underlying GHB/GBL dependence and wishes to undergo GHB/GBL detoxification or is thought to have underlying GHB/GBL dependence and is at risk of acute withdrawal and, for the outpatient arm only, who is registered with a drug treatment service, will be eligible for the study.

#### Exclusion criteria

An individual will not be eligible for inclusion in this study if they are unable to provide written informed consent and any of the following criteria apply: the clinician decides that medication is not required for the management of GHB/GBL withdrawal; if medication is indicated but the patient lacks capacity to consent, is unable to take oral medication or is unable to take baclofen according to SPC due to known hypersensitivity to baclofen or any of the excipients, hereditary problems of galactose intolerance, the Lapp lactase deficiency or glucose-galactose malabsorption, active peptic ulceration or porphyria. In addition, for those with epilepsy that is not well-controlled, either with or without medication, or those with end-stage renal failure (Chronic Kidney Disease (CKD) stage 5, GFR below 15 mL/min) (which have special warnings and precautions for use, according to the SPC) will not be entered since the risk versus benefit ratio for prescribing is not in favour of prescribing baclofen. In addition, participants who are unable to follow the study protocol due to serious mental health disorder, e.g. enduring psychotic illness or suicidal intent will not be eligible. Further, any participant who has taken any investigational drug within 30 days prior to drug administration or any woman refusing a pregnancy test will not be eligible.

### Randomisation

An assessment of eligibility will be made by a physician following discussion with the potential participant and review of the accessible medical records. For some participants wishing to participate in the study but where there is limited or no access to medical records, the individual will be included in the trial unless one or more of the eligibility criteria are known not to be met. Informed consent will be obtained by a member of the study team.

To be able to respond to the urgent needs of people who are at high risk of withdrawing from GHB/GBL in a timely manner, the study medication will be pre-packed and pre-randomised and stored at both study sites. Randomisation codes in blocks of 4 (for the planned withdrawal group) and blocks of 15 (for the unplanned withdrawal group) will be generated by the trial statistician. Those eligible for inclusion will be randomised to baclofen or placebo by taking the next lowest consecutively numbered pack from storage cupboards in the respective treatment centre.

**Fig. 1:**
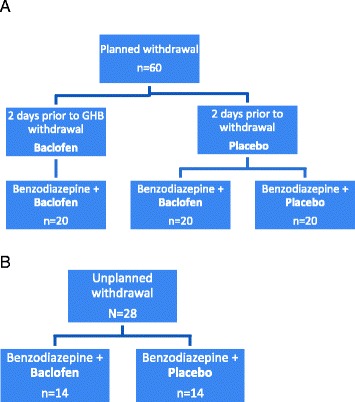
**a** CNWL Club Drug Clinic – Planned (outpatient) withdrawal, *N* = 60 (three groups). **b** GSTT clinical toxicology service – Unplanned (inpatient) withdrawal, *N* = 28 (two groups). *CNWL* Central North West London NHS Foundation Trust, *GSTT* Guy’s and St. Thomas’s NHS Foundation Trust

If eligible, have given informed consent and following baseline assessment, the participants are allocated as follows (see Fig. 1a and b). Those in the planned arm will receive a preload before their detoxification proper and will be allocated to one of three groups: preload of placebo (one tablet three times a day) followed by the benzodiazepine GHB/GBL detoxification regimen with the study medication, baclofen (10 mg three times a day) or placebo (one tablet three times a day) for up to 10 days, or preload of baclofen (10 mg three times a day) followed by the benzodiazepine GHB/GBL detoxification regimen with baclofen (10 mg three times a day) for up to 10 days. Those in the unplanned arm will receive their benzodiazepine detoxification regimen for GHB/GBL withdrawal with the study medication, baclofen (10 mg three times a day) or placebo (one tablet three times a day) for up to 10 days. In both unplanned and planned GHB/GBL withdrawal, clinicians will use the benzodiazepine and dosing regimen that they consider appropriate. The study medication will be discontinued at the same time as the benzodiazepine, with the decision about when both medications are to be discontinued made by their clinician.

### Assessment and outcome measures

Data will be collected to inform the primary and secondary outcome measures in the definitive trial (see Table 1). The proposed primary outcome measures include symptom severity, complications and requirement for treatment escalation, and the proposed secondary outcomes include change in anxiety, depression, sleep and quality of life as well as relapse to GHB/GBL use or change in other illicit drug or alcohol use 30 days after the start of detoxification.

**Table 1:** Assessment schedule of quantitative measures

Assessment	Baseline or as soon as data can be obtained	Daily during detoxification			Last day of detoxification or next working day	Ad hoc	30 days post start of detoxification
		Inpatient	Weekday outpatient	Weekend outpatient			
CIWA-Ar	✓	✓	✓	✓	✓		
Sedation Assessment Tool	✓	✓	✓	✓			
Depression (PHQ-9)	✓				✓		
Anxiety (GAD-7)	✓				✓		
Sleep pattern questionnaire	✓	✓	✓	✓	✓		
Vital signs	✓						
Psychiatric history	✓						
Relevant past medical history	✓						
Concomitant prescribed medication	✓	✓	✓	✓	✓		
Lifetime GHB/GBL use questionnaire					✓		
Daily drug/alcohol/nicotine use questionnaire	✓	✓	✓	✓			
Time-life follow-back of alcohol and drug use					✓		
AUDIT (alcohol)					✓		
Treatment Outcome Profile					✓		
Client Satisfaction Questionnaire (CSQ-8)					✓		
Adverse events		✓	✓	✓		✓	
Expected events associated with GHB/GBL withdrawal						✓	
Protocol deviations						✓	
Follow-up GHB/GBL use questionnaire							✓
Follow-up drug/alcohol use questionnaire							✓
Follow-up service use questionnaire							✓

*AUDIT* Alcohol Use Disorders Identification Test, *CIWA-Ar* Clinical Institute Withdrawal Assessment for Alcohol, *GAD-7* Generalised Anxiety Disorder-7, *GBL* gamma-butyrolactone, *GHB* gamma-hydroxybutyrate, *PHQ-9* Patient Health Questionnaire-9

On the first day of GHB/GBL detoxification, ratings will be obtained from each participant of their symptoms of GHB/GBL withdrawal using a scale widely used for alcohol withdrawal – the Clinical Institute Withdrawal Assessment for Alcohol (CIWA-Ar) which is currently used clinically by teams to monitor GHB/GBL withdrawal [32–35]. The CIWA-Ar may not adequately capture all neuropsychiatric symptoms/signs [32, 33] and the Sedation Assessment Tool (SAT) will assess behavioural disturbance [36]. Depressive (Patient Health Questionnaire-9 (PHQ-9); [37]) and anxiety (Generalized Anxiety Disorder-7 (GAD-7); [38]) symptoms will be assessed using scales that are already in routine clinical use. We will also record sleep pattern using a questionnaire [39]. Any medication taken by the time of the ratings will be recorded. Blood pressure, respiratory rate, oxygen saturation and pulse will also be recorded when the CIWA-Ar is completed. Since this is a pragmatic trial, we aim to minimise any extra tests or investigations and research participants will receive standard medical care as usual. If, as part of this standard medical care, blood and/or urine will be collected for toxicological screening for recreational drugs/novel psychoactive substances, the results will be recorded but not sought solely for the purposes of this study.

During detoxification, daily assessments will record the withdrawal assessment scale (CIWA-Ar; [29]), behavioural disturbance (SAT; [36]), sleep pattern, any use of other drugs/novel psychoactive substances/alcohol and nicotine (including substitution), as well as when they took any medication. On the last day of taking detoxification medication (or the next working day for outpatients where this falls on a weekend/public holiday), withdrawal, depressive and anxiety symptoms (PHQ-9: [37]; GAD-7: [38]) and sleep pattern will be recorded for all participants. Research participant satisfaction will be measured with the Client Satisfaction Questionnaire-8 (CSQ-8; [40, 41]). Any information not already obtained or previously available will also be completed and will include the following: using a semistructured questionnaire: years of GHB/GBL use, the amount of GHB/GBL used in the week prior to detoxification and the amount in a typical day/week, pattern of use, whether they are self-reported ‘dependent’ on any of these medication(s) that have been used to help self-treat or prevent previous withdrawal. Previous psychiatric and medical history, other drug or alcohol use including nicotine and determination of any dependence will be similarly established as well as using time-life follow-back, the alcohol screening questionnaire (Alcohol Use Disorders Identification Test (AUDIT); [42]) and appropriate parts of the mandatory Treatment Outcomes Profile (TOP; [43]) used in substance misuse services will be completed.

Regarding medication, the total amount of benzodiazepine prescribed, its dosing regimen and quantity taken will be recorded. Use of any other medication during the detoxification will also be recorded.

Any complications arising during preload or detoxification will be recorded. These will be classified as adverse events if they are not one of the following predetermined events which are known to occur in acute GHB/GBL withdrawal or detoxification: agitation or aggression, hallucinations (visual, auditory and/or tactile), tachycardia, seizures, ataxia, sedation, tremor and/or sweating. All adverse events occurring from the time a participant signs the Consent Form until completion of the last study-related procedure will be recorded in the Case Report Form. Any serious adverse event which occurs in a participant will be reported immediately to the chief investigator (CI) and sponsor and, if stated orally, will be followed by a detailed written report.

On day 30 after the start of detoxification (benzodiazepine detoxification, not preload), the researcher will contact the research participant to obtain information about any subsequent use of GHB/GBL, other recreational drugs/novel psychoactive substances/alcohol as well as any medication (prescribed or otherwise obtained) taken and psychosocial treatment/support received for their GHB/GBL dependence. In addition, any issues or events that could be related to their detoxification will be recorded.

Patients will be asked to return any remaining study medication to the clinic so that a pill count can be performed to assess compliance.

### Study medication discontinuation and withdrawal from the study

In accordance with the current revision of the Declaration of Helsinki (amended October 2000, with additional footnotes added in 2002 and 2004), a participant has the right to stop trial treatment and to withdraw from the trial at any time and for any reason, without prejudice to his or her future medical care by the physician or at the institution, and is not obliged to give his or her reasons for doing so. The principal investigator’s clinical judgement will determine whether or not an adverse event is of sufficient severity to require discontinuation of the study treatment. A participant who discontinues the study medication before the end of their detoxification will not be withdrawn from the study. Provided they have not withdrawn their consent, they would continue to be followed up to enable an intention-to-treat analysis. In addition, as this is a feasibility study, they may be approached for the qualitative study.

### Study unblinding

Masking of treatment allocation will be maintained during an individual’s participation in the trial unless any of the following occur: a serious adverse event arises that clinically requires disclosure or another clinical reason to need to know the allocation such as to start the participant on medication which has a risk of interaction. The 24-h emergency unblinding service will allow a medical request for unblinding in the event of a medical emergency. Procedures will be put in place to verify the identity of the participant and caller, and the decision on whether to reveal the study medication allocation will be based on a set of criteria for judging clinical need. All requests for unblinding will be recorded.

### Sample size

The aim is to recruit 88 research participants who are undergoing GHB/GBL detoxification: 60 planned outpatients and 28 unplanned inpatients.

### Qualitative process evaluation: research participant and clinician acceptability

We will undertake qualitative, semistructured, one-to-one interviews with 12 research participants, 6 from each site. Informed consent will be separately sought for this aspect of the study. Participants will be sampled purposively to represent key case-mix variables and treatment allocation. These interviews will investigate (1) participants’ experience and acceptance of the study procedures (notably recruitment, randomisation and outcome assessment procedures) and (2) their treatment experience. The interviews will be conducted using a topic guide. This will be drafted on the basis of the study aims, relevant scientific literature and clinical experience, but there will be scope for iterative development of the guide as data collection and analysis progress. Participants who have undergone a previous detoxification will be asked to reflect on how the trial detoxification compared with previous ones.

In addition, two focus groups involving the clinical teams from each site will also be conducted. These will explore clinician experience of the trial and the acceptability of trial procedures. Key senior members of staff will also be offered a one-to-one semistructured interview. All interviews and focus groups will be audio-recorded, transcribed and analysed using a thematic framework approach and managed using NVivo (Scolari/Sage) computer software. Initial coding frameworks for both participant and staff datasets will be based on the study aims of how feasible and acceptable it is to undertake a definitive trial, but subthemes will be further developed through analytic induction and grounded in the data. This will be revised iteratively as data collection and analysis progress.

### Cost-effectiveness

Data will be collected to inform the undertaking of a full economic evaluation in the definitive randomised controlled trial (RCT) by piloting the data collection methods that would be used in such a full trial. Piloting data collection instruments will show the feasibility of recording service use information from the population and identify the services and other costs to participants that would need to be included in a full economic evaluation of a phase III trial.

### Safety monitoring plan

An Independent Data Monitoring and Ethics Committee (IDMEC) will be established to provide overall supervision of the trial and ensure that it is being conducted in accordance with the principles of Good Clinical Practice (GCP) and the relevant regulations. It will report to the Trial Steering Committee which provides overall supervision for the GHB trial on behalf of the trial sponsor and the trial funder, ensures that the trial is appropriately conducted, and provides advice, through its chair, to the Trial Management Group.

The end of the trial is defined as completion of follow-up of, or three attempts to contact, the last participant randomised to the study medication. The trial may terminate before all (*n* = 88) participants have been recruited if we have obtained sufficient information about recruitment, engagement and/or evaluation of outcome measures.

The NHS Indemnity Scheme applies and provides unlimited cover for NHS staff, medical academic staff with honorary contracts and those conducting research, for negligent harm. Non-negligent harm (i.e. harm that has been caused through no fault of those conducting research) is not covered by this scheme; however, ex gratia payments may be considered by the trust in limited circumstances.

### Trial monitoring

Each site in the trial will receive two on-site monitor visits, firstly when five participants have been randomised to the trial and a second site visit 6–9 months from the time of site activation. Additional monitor visits will be conducted if required. These will be conducted by the sponsor, CI and trial manager.

### Data

The anonymised data will be stored on a laptop and backed up on a secure server. The personal datasheet and code associated with that person will be kept locked up. All data handling and record keeping will adhere to the Data Protection Act 1998. Regarding access to source data, the sponsor and investigators will permit monitoring, audits, Research Ethics Committee (REC) and Medicine and Healthcare products Regulatory Agency (MHRA) review (as applicable) and provide direct access to source data and documents as appropriate.

### Data analysis

The primary focus of this study is to assess the feasibility of undertaking a definitive trial. In particular, we will characterise the optimal recruitment rate and strategies and characteristics of the proposed primary outcome measures (symptom severity, complications, and requirement for treatment escalation). The main analyses will be descriptive and provide estimates of variability and effect size with 95 % confidence intervals. This will include the rate of recruitment, the proportion of people approached who consent to randomisation, the proportion who complete detoxification and the proportion who will complete the follow-up assessment. We will also describe the distribution of scores on the primary outcomes and estimate the variance of the measure in this population. Data for our proposed secondary outcomes in the definitive trial will include change in anxiety, depression, sleep and quality of life as well as relapse to GHB/GBL use or change in other illicit drug or alcohol use 30 days after the start of detoxification. We will also identify appropriate criteria, e.g. benzodiazepine requirement or withdrawal score for minimisation or stratification in the full RCT.

We will use ANCOVA adjusting for patients’ characteristics and relevant clinical data. Where there is follow-up, methods applied to longitudinal data will be appropriate since they are able to detect significant differences between arms but they can also detect changes of the outcome measure(s) over time. We will also conduct an interim analysis to characterise the number of adverse events in each arm as described above.

An interim analysis to characterise the number of adverse events in each arm will also be conducted. Treatment escalation (i.e. admission to ICU for those presenting to the ED (unplanned) and admission to the general hospital for those in the Club Drug Clinic (planned)) will be closely monitored. From our clinical experience of complications from GHB/GBL withdrawal, if five individuals in any one group require treatment escalation, the sponsor and IDMEC will be immediately informed and recruitment suspended until there has been a discussion and consideration of the protocol and trial continuing. Stopping rules will also be agreed which specify the point at which interim results will be judged to be sufficiently conclusive for it to be appropriate for the IDMEC to recommend that they consider early termination of the trial.

### Trial dissemination

The outcome of the trial will be communicated to the clinical and academic communities through presentations at conferences and meetings including those attended by users, and publications in peer-reviewed journals and general media if appropriate. These will be completed by members of the study team. These will be in line with NIHR publication procedures.

## Discussion

Whilst GHB/GBL may not be widely used by the general population, there is greater use in certain subpopulations such as gay and bisexual males (GBM). Regular use of GHB and its related analogues can lead to the development of physical dependency and an associated withdrawal syndrome on stopping use [1, 2]. This typically is rapid in onset after last dose and may mimic other conditions (e.g. acute stimulant toxicity, acute alcohol withdrawal) making its diagnosis and management challenging for clinicians [2, 11, 14]. This is particularly of concern in areas where GHB and related analogue use is low, so there may be a lack of awareness of the potential for dependency and withdrawal. A proportion of individuals with GHB-related withdrawal may require more intensive sedation and subsequent admission to HDU/ICU to ensure not only their safety, but also that of the staff caring for them. This incurs significant costs to the individual through length of stay and potential for ICU-related complications, as well as to the NHS and wider society due to the required resource utilisation.

There is much anecdotal evidence that the current clinical practice of using benzodiazepines alone as initial treatment may be insufficient to adequately manage GHB/GBL withdrawal and prevent the requirement for treatment escalation. Since GHB/GBL acts as a GABA_B_ receptor agonist, using another GABA_B_ agonist, baclofen, to attenuate withdrawal has pharmacological validity. Our clinical experience, and that of others, is that the addition of baclofen (10 mg three times a day) to benzodiazepines does indeed reduce GHB/GBL withdrawal symptoms and its associated complications [11, 14]. Furthermore, preloading with baclofen for 2 days prior to detoxification appears to provide additional benefits. When designing this trial, we were not aware that this is undertaken routinely outside our specialist clinic and it is important to understand its role in improving and managing GHB/GBL withdrawal. A similar approach may be used by some clinicians for particular patients to help them to prepare for their alcohol detoxification by reducing cravings and excessive consumption prior to starting their detoxification proper.

Due to its use in particular communities, specific addiction services have evolved with expertise in managing GHB/GBL withdrawal. Similarly, presentations to EDs vary depending on local clubs and nightlife, since individuals who frequent these tend to also live in the local area. The two services in this study have such local communities and have developed their services to meet the local needs. They are, therefore, well-placed to conduct this study due to their experience and the regular presentations of those with GHB/GBL withdrawal. There is local support and much user interest in this study.

In order to determine whether baclofen is a useful adjunct and to meet thresholds required by national guidelines, e.g. NICE, a trial is still required; without such a trial the use of baclofen will remain based on anecdotal evidence. We are not aware of any other trial using baclofen in GHB withdrawal nor has any other pharmacological approach been shown to be robustly effective or appropriate for community-based detoxification. Importantly this trial also seeks to establish whether the use of baclofen prior to starting detoxification, i.e. a preload improves symptom control and reduces complications during GHB detoxification. This is a novel aspect of the study and addresses a critical question.

## Trial status

The study has received favourable opinions from the MHRA and the REC. The aim is to start recruitment by Spring 2016.

## Abbreviations

AUDIT: Alcohol Use Disorders Identification Test
CIWA-Ar: Clinical Institute Withdrawal Assessment for Alcohol
CNWL: Central North West London NHS Foundation Trust
ED: Emergency department
EMCDDA: European Monitoring Centre for Drugs and Drug Addiction
GBL: Gamma-butyrolactone
GCP: Good Clinical Practice
GHB: Gamma-hydroxybutyrate
GMP: Good Manufacturing Practice
GSTT: Guy’ and St. Thomas’s NHS Foundation Trust, London
ICU: Intensive care unit
IDMEC: Independent Data Monitoring and Ethics Committee
RCT: Randomised controlled trial
SPC: Summary of Product Characteristics
